# Coronary Artery Disease Evaluation in Rheumatoid Arthritis (CADERA): study protocol for a randomized controlled trial

**DOI:** 10.1186/1745-6215-15-436

**Published:** 2014-11-08

**Authors:** Bara Erhayiem, Sue Pavitt, Paul Baxter, Jacqueline Andrews, John P Greenwood, Maya H Buch, Sven Plein

**Affiliations:** Multidisciplinary Cardiovascular Research Centre & Leeds Institute for Cardiovascular and Metabolic Medicine, University of Leeds, Worsley Building, Clarendon Way, Leeds, LS2 9JT UK; Leeds Institute of Health Sciences, University of Leeds, Charles Thackrah Building, 101 Clarendon Road, Leeds, LS2 9LJ UK; Division of Epidemiology & Biostatistics, Leeds Institute for Cardiovascular and Metabolic Medicine, University of Leeds, Worsley Building, Leeds, LS2 9JT UK; Leeds Institute of Rheumatic and Musculoskeletal Medicine, Chapel Allerton Hospital, 2nd Floor, Chapeltown Road, Leeds, LS7 4SA UK; National Institute for Health Research Leeds Musculoskeletal Biomedical Research Unit, Chapel Allerton Hospital, Leeds Teaching Hospitals NHS Trust, Leeds, LS7 4SA UK

**Keywords:** Cardiovascular magnetic resonance, Rheumatoid arthritis, Biological therapy, Etanercept, Methotrexate, Coronary artery disease, Aortic distensibility, MOLLI, Perfusion CMR, Late gadolinium enhancement

## Abstract

**Background:**

The incidence of cardiovascular disease (CVD) in rheumatoid arthritis (RA) is increased compared to the general population. Immune dysregulation and systemic inflammation are thought to be associated with this increased risk. Early diagnosis with immediate treatment and tight control of RA forms a central treatment paradigm. It remains unclear, however, whether using tumor necrosis factor inhibitors (TNFi) to achieve remission confer additional beneficial effects over standard therapy, especially on the development of CVD.

**Methods/Design:**

Coronary Artery Disease Evaluation in Rheumatoid Arthritis (CADERA) is a prospective cardiovascular imaging study that bolts onto an existing single-centre, randomized controlled trial, VEDERA (Very Early versus Delayed Etanercept in Rheumatoid Arthritis). VEDERA will recruit 120 patients with early, treatment-naïve RA, randomized to TNFi therapy etanercept (ETN) combined with methotrexate (MTX), or therapy with MTX with or without additional synthetic disease modifying anti-rheumatic drugs with escalation to ETN following a ‘treat-to-target’ regimen. VEDERA patients will be recruited into CADERA and undergo cardiac magnetic resonance (CMR) assessment with; cine imaging, rest/stress adenosine perfusion, tissue-tagging, aortic distensibility, T1 mapping and late gadolinium imaging. Primary objectives are to detect the prevalence and change of cardiovascular abnormalities by CMR between TNFi and standard therapy over a 12-month period. All patients will enter an inflammatory arthritis registry for long-term follow-up.

**Discussion:**

CADERA is a multi-parametric study describing cardiovascular abnormalities in early, treatment-naïve RA patients, with assessment of changes at one year between early biological therapy and conventional therapy.

**Trials registration:**

This trial was registered with Current Controlled Trials (registration number: ISRCTN50167738) on 8 November 2013.

## Background

Rheumatoid arthritis (RA) is one of the most common autoimmune diseases affecting approximately 1% of the population in the United Kingdom [1]. RA is a chronic, systemic inflammatory arthritis and, if not adequately controlled, can lead to significant joint damage and subsequent functional impairment. Mortality is increased up to three-fold compared to the general population, largely due to increased frequency of premature cardiovascular disease (CVD), which causes up to 40% of mortality cases in RA patients [2], and is as high as that of patients with other major CVD risk factors such as type 2 diabetes mellitus [3]. It is accepted that CVD risk in RA is independent of, and incremental to, traditional CVD risk factors [4], with the likely predominant pathological process being immune dysregulation leading to systemic inflammation [5], however the exact mechanisms remain unclear. The inflammatory process, mediated through pro-inflammatory cytokines such as tumor necrosis factor (TNF), is linked to atherosclerosis and plaque rupture and has confounding effects on lipid and glucose metabolism, blood pressure and hemostatic factors [6]. Markers of RA severity are strongly associated with adverse cardiovascular (CV) outcomes in RA [7], with atherosclerosis itself being increasingly viewed as an inflammatory-mediated process [8].

Arterial stiffness is associated with an increased risk of CV events with a range of co-morbidities [9]. In patients with RA without traditional CV risk factors, aortic pulse wave velocity is higher than in controls [10] and correlates with age, mean arterial pressure and C-reactive protein (CRP). Echocardiography studies have shown that patients with RA have high rates of diastolic dysfunction [11], heart failure [12, 13] and heart failure with preserved ejection fraction (EF) [14]. Positron emission tomography (PET) in patients with rheumatic diseases without coronary artery disease (CAD) shows lower myocardial blood flow (MBF) reserve compared to controls, with an inverse correlation to disease duration [15]. In a meta-analysis of 22 studies, RA patients had a greater carotid intimal-media thickness ((CIMT) a direct measure of the status of the vascular wall and measure of atherosclerotic and arteriosclerotic processes [16]) than controls [17], with emerging evidence that CIMT is abnormal even in early disease [18]. These findings are consistent with the concept of microvascular pathology and accelerated atherosclerosis due to systemic inflammation in RA, which may precede and contribute to the effects of CAD.

Early diagnosis of RA and immediate intervention with conventional disease modifying anti-rheumatic drugs (DMARDs) in a treat-to-target approach, with remission the goal of treatment, is an internationally recommended, established practice [19]. Biological DMARD (bDMARD) treatments, first introduced at the turn of the century, are highly effective tools to achieve this and have revolutionized outcomes in RA. The TNF-inhibitors (TNFi) were the first bDMARD agents to be introduced, applied in the methotrexate (MTX) failure population, with remarkable structural benefits also observed. More recently however, first-line TNFi studies in early RA have demonstrated particularly high rates of remission induction, similar or slightly greater than conventional DMARD, but with superior structural benefits and the ability to achieve drug-free remission [20–26]. In addition, reports have suggested wider benefits of bDMARD therapy including reduction in biomarkers associated with CVD [27, 28]. Recent pilot data has shown that tocilizumab treatment for over one year significantly increased left ventricular ejection fraction and decreased left ventricular mass index associated with disease activity [29].

CV clinical trials of TNFi treatments in RA are challenging because of the small number of hard clinical CV mortality endpoints in study populations [30], and being unable to adjust for important confounders that differentiate between CV events that follow other pathophysiological pathways [31]. As TNFi treatment is reserved for patients with established, MTX-resistant diseases, observational studies are inherently limited by a selection bias. Although aggressive treat-to-target approaches with conventional DMARDs are associated with impressive remission rates, the use of bDMARD may offer a ‘window of opportunity’ in early RA by interrupting progression along the disease continuum, and consequently may have the additional potential to impact CVD.

### Detection of cardiovascular disease in rheumatoid arthritis

The imaging modalities currently used for the assessment of CVD in RA are transthoracic echocardiography (TTE), single-photon emission computed tomography (SPECT) and cardiovascular magnetic resonance (CMR) [32]. PET is recognized as the gold standard for MBF quantification but is hindered by high cost and low availability and offers little functional information. SPECT is commonly used for ischemia testing but, as with PET, it cannot assess cardiac structure and exposes patients to a significant dose of ionizing radiation [33]. TTE is a safe, low-cost examination that can assess cardiac structure and function and provides information on ischemia and viability when combined with exercise and/or pharmacological stress. Poor acoustic windows can be a common problem due to obesity or acoustic shadowing from the lungs and reporting variability limits its reproducibility.

### Cardiovascular magnetic resonance

CMR is widely recognized as a safe, sensitive, reproducible and comprehensive non-invasive imaging test to detect CVD. Both anatomical and functional assessment can be made with CMR. Left ventricular (LV) mass and function can be measured more accurately than with any other imaging method [34]. Aortic distensibility can be reliably measured from the ascending or descending aorta [35]. Tissue tagging provides measurements of regional and global myocardial strain as an early marker of contractile dysfunction [36]. We have shown in a large study of patients with suspected angina that CMR can detect myocardial ischemia with greater sensitivity than nuclear perfusion imaging [37]. Dynamic contrast enhanced CMR methods combined with quantitative analysis can be used to estimate MBF at rest and during hyperemic stress [38]. Perfusion CMR has demonstrated reduced MBF reserve in asymptomatic adults with CVD risk factors, suggesting it can detect preclinical pathology [39]. T1 mapping methods are used to measure the extent of the extracellular matrix in the heart, which expands in response to inflammation and fibrosis [40]. CMR has no harmful effects and multiple measurements can be combined in a single imaging protocol [35].

The literature on CMR in RA is sparse. In contrast to previous TTE studies, CMR shows that patients with RA have reduced LV mass and EF [41]. No previous studies have combined macrovascular, microvascular and detailed myocardial assessment by CMR in RA, such that the full potential of CMR for a comprehensive multi-parametric and quantitative evaluation of CVD in RA has not yet been realized.

### Hypotheses

We hypothesize that the CADERA study will determine, using multi-parametric CMR, that i) subclinical CV pathology exists in patients with early, treatment-naïve RA, ii) early aggressive control of RA can reduce this subclinical CV pathology at one year from treatment initiation and iii) TNFi offer additional benefit over and above conventional DMARD in the burden of subclinical CV pathology.

## Methods/Design

### Study design

**Figure 1:**
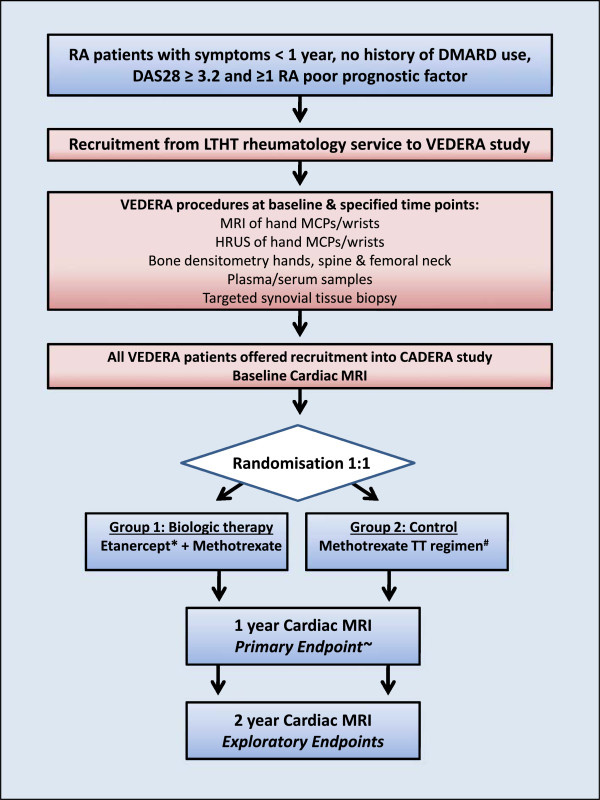
**Coronary Artery Disease Evaluation in Rheumatoid Arthritis (CADERA) study flow diagram.** *Etanercept non-responders or intolerance managed at physician’s discretion. ^#^Methotrexate for duration of study, addition of other DMARDs at week eight if not in remission and escalated to etanercept at week 24 if not in remission. ^~^Etanercept discontinued at the primary endpoint unless clinically indicated and at physician’s discretion. DAS, disease activity score; DMARD, disease modifying anti-rheumatic drug; HRUS, high-resolution ultrasound; LTHT, Leeds Teaching Hospitals NHS Trust; MCP, metacarpophalangeal; RA, rheumatoid arthritis; TT, treat-to-target; VEDERA, Very Early versus Delayed Etanercept in Rheumatoid Arthritis.

CADERA bolts on to the VEDERA (Very Early versus Delayed Etanercept in Rheumatoid Arthritis) trial, a prospective longitudinal intervention study of patients with early RA, randomized to either first-line TNFi therapy (etanercept, ETN) and MTX or optimal synthetic DMARD therapy. VEDERA is an investigator-initiated research (IIR) study based at the Leeds Institute of Rheumatic and Musculoskeletal Medicine, and is funded by an unrestricted educational grant that is part of an IIR agreement with Pfizer. VEDERA is a phase IV, single-centre study of 120 patients with new-onset, treatment-naïve RA, randomized to either immediate ETN and MTX combination or initial MTX and a treat-to-target regimen (optimal, standard conventional therapy approach); with step-up in the latter group to ETN and MTX combination therapy in patients failing to achieve a pre-defined target of remission after 24 weeks. The aim of VEDERA is to assess for the depth of remission (clinical and imaging) and immunological normalization induced by the treatment arms, as well as to identify predictors of remission.VEDERA patients will be recruited to CADERA and undergo CMR at baseline (prior to treatment) as well as after one and two years of treatment (see Figure 1). The change in CVD status as defined by CMR between baseline and follow-up in patients treated with early biological or optimal DMARD therapy will be determined. The study flow chart is presented in Figure 1. At the end of the study all patients will enter an inflammatory arthritis registry based at the National Institute for Health Research (NIHR) Leeds Musculoskeletal Biomedical Research Unit (LMBRU).

The National Research Ethics Service Committee Yorkshire and The Humber - Leeds West has approved the study protocol and other relevant documentation (Research Ethics Committee reference: 10/H1307/138).

### Enrolment criteria

Patients eligible for VEDERA will be recruited from the Leeds Teaching Hospitals NHS Trust Rheumatology service. The recruitment period is expected to last up to 36 months. All patients recruited to VEDERA will be offered inclusion to the CADERA study. CADERA CMR scans will be performed and analyzed at Leeds General Infirmary. The study will be performed in accordance with the Declaration of Helsinki (October 2000), with all patients providing informed written consent.

Inclusion criteria for VEDERA, and therefore CADERA, are patients diagnosed with RA according to the 2010 American College of Rheumatology/The European League Against Rheumatism (ACR/EULAR) criteria (Table 1), who have not yet received therapy with DMARDs, have early (symptoms for less than one year) active disease (clinical or imaging evidence of synovitis and Disease Activity Score in 28 joints with Erythrocyte Sedimentation Rate (DAS28-ESR) ≥3.2) and at least one poor prognostic factor (anti-citrullinated peptide antibody (ACPA) +/- abnormal power doppler in at least one joint).

**Table 1:** **The 2010 ACR/EULAR classification criteria for rheumatoid arthritis**

Criteria	Score
Joint distribution	
1 large joint	0
2-10 large joints	1
1-3 small joints (large joints not counted)	2
4-10 small joints (large joints not counted)	3
>10 joints (at least one small joint)	5
Serology	
Negative RF AND negative ACPA	0
Low positive RF OR low positive ACPA	2
High positive RF OR high positive ACPA	3
Symptom duration	
<6 weeks	0
≥6 weeks	1
Acute phase reactants	
Normal CRP AND normal ESR	0
Abnormal CRP OR abnormal ESR	1

A score of six or more equates to definite RA. This requires that the patient has at least one joint with definite synovitis and that the synovitis is not better explained by another disease. The score may be retrospective or prospective. ACPA, anti-citrullinated peptide antibody; CRP, C-reactive protein; ESR, erythrocyte sedimentation rate; RF, rheumatoid factor.

Exclusion criteria are previous treatment with DMARDs, known CVD, contraindications to TNFi therapy (or severe co-morbidity that would in the clinician’s opinion be associated with unacceptable risk of receiving TNFi therapy) and contraindications to CMR, (which include renal failure (estimated Glomerular Filtration Rate (eGFR) <30 ml/min/1.73 m^2^), known allergy to gadolinium-based contrast agents and contraindications to adenosine (asthma or high-grade heart block)).

### Primary outcome measure

The primary outcome measure is aortic distensibility. It will be measured and quantified at baseline and at one year in each arm of the study. Increased arterial stiffness is associated with an increased risk of CV events [9]. It can be measured by pulse wave velocity or as distensibility of the aorta, but requires careful correction for age and blood pressure. It has previously been shown that aortic distensibility relates to clinical outcome and that TNFi improve aortic distensibility [27]. We performed a pilot study in 10 patients with RA (disease duration 20 ± 9.6 years) and matched by age and gender to 10 asymptomatic subjects without RA. Aortic distensibility was significantly different in RA patients, with a mean and standard deviation of 1.83 ± 0.4 cm^2^ versus 2.6 ± 0.6 cm^2^ in controls. LV volumes and mass were similar between groups and LV strain and twist showed trends towards a reduction in RA patients, but without reaching statistical significance. Our pilot data therefore suggested CV abnormalities in patients with RA in several quantitative CMR parameters, with aortic distensibility reaching statistically significant difference even in the small sample size.

Longitudinal changes of outcome measures in response to therapy will be measured and compared between the two treatment arms at baseline, one and two year time points. Secondary outcome measures are i) myocardial perfusion reserve, ii) LV strain and twist, iii) LVEF and iv) LV mass. Exploratory outcome measures are pre- and post-contrast T1 mapping, extra-cellular volume (ECV) and biomarker measurements.

Significant differences (expressed as *P* <0.05) of CV abnormalities detected by CMR between the two treatment arms will be presented, and the magnitude of this difference will be expressed as a 95% confidence interval.

### Sample size calculation

Power calculations are based on a previous study by Ikonomidis *et al*. [28]. We assumed an effect size of 2.46 cm^2^dyne^-1^10^-6^, representing 75% of the difference between treated (Anakinra) and non-treated RA patients reported by Ikonomidis *et al*. [28]. Mean aortic distensibility at baseline to post-treatment for treated and non-treated patients was 1.56 cm^2^dyne^-1^10^-6^ and 4.6 cm^2^dyne^-1^10^-6^, respectively. The standard deviation (SD) of the post-treatment measurements in the Anakinra group was 3.2 cm^2^dyne^-1^10^-6^ and a more conservative estimate of 3.5 cm^2^dyne^-1^10^-6^ has been used in the CADERA power calculation. Assuming an SD of 3.5 cm^2^dyne^-1^10^-6^, a power of 70%, 80% and 90% would be achieved at 5% significance level in a two-tailed independent samples Student’s t-test with 26, 33 and 44 patients respectively in the primary outcome measure of aortic distensibility in each treatment group (30, 38 and 50 when adjusted for 10% dropout).

Both treatment arms will be compared with primary outcome aortic distensibility from baseline to one-year follow-up, as well as other outcome measures. Analysis will be conducted in the R environment for statistical computing (R Core Team, 2012. R: A language and environment for statistical computing. R Foundation for Statistical Computing, Vienna, Austria). Exploratory data analysis will be used to determine if parametric (independent samples Student’s t-test) or non-parametric (Wilcoxon rank sum test) analyses are appropriate, and to summarize the distribution of aortic distensibility and change in other outcome measures across the two treatment arms. These analyses will also allow the credibility of an equal variance assumption to be assessed in parametric modeling and to be appropriately modeled [42]. All patients meeting eligibility criteria will be included in the analyses and these will be conducted at the end of the recruitment period. Exploratory subgroup analyses will be conducted separately by other comorbidities, a maximum of two to three that are clinically plausible, with appropriate correction for multiple testing [43]. Interactions between subgroups and interactions between CMR findings and biomarkers will be explored through building a linear model with interaction terms [44]. Patterns of CVD pathology in RA patients will be described. Treatment effects on secondary outcome measures and effects at the two-year follow-up point will be analyzed in an equivalent manner.

#### Missing data

The numbers of patients with missing data for one or more CMR measurements, and the number of uninterpretable images will be reported. Patients with missing data for any CMR measurement will be excluded from any comparison involving that measurement.

#### Test conduct

The number of patients referred from VEDERA and failing to complete the CMR protocol will be reported, along with the reason why the test failed. The duration of the CMR scan will also be summarized.

### Cardiac magnetic resonance investigation details

**Figure 2:**
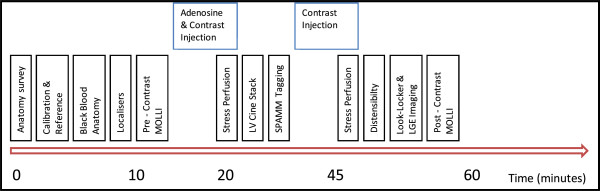
**Coronary Artery Disease Evaluation in Rheumatoid Arthritis (CADERA) cardiac magnetic resonance protocol.** LGE, late gadolinium enhancement; LV, left ventricular; MOLLI, modified Look-Locker inversion method; SPAMM, spatial modulation of magnetization.

Our group has well-established multi-parametric protocols that have been validated in other populations [45]. CMR will be performed on a dedicated 3 T Philips Achieva TX system equipped with a 32-channel coil, vectorcardiographic triggering and multi-transmit technology (Philips Healthcare, Best, The Netherlands). Patients will be asked to avoid caffeine for 24 hours prior to the scan. The CMR protocol (Figure 2) lasts approximately 60 minutes and will comprise of:

1. Low-resolution survey, reference scans and localizers. Following survey and reference scans, the heart’s short axis, vertical long axis and horizontal long axis will be defined with a series of cine images (balanced steady-state free precession acquisition (bSSFP), echo time (TE) 1.48 ms, repetition time (TR) 3.0 ms, flip angle 45°, field of view 320 to 420 mm according to patient size, slice thickness 10 mm and 30 phases per cardiac cycle).
2. Baseline T1 mapping. One slice will be acquired at the LV short axis using an electrocardiogram (ECG)-triggered modified Look-Locker inversion (MOLLI) method to acquire 11 images (3-3-5 acquisition with 3 × R-R interval recovery epochs) in a single end-expiratory breath hold (voxel size 1.7 × 2.14 × 10 mm^3^ trigger delay at end-diastole, flip angle 35° and field of view 320 to 420 mm) [46, 47].
3. Adenosine stress first-pass myocardial perfusion imaging (spoiled Turbo Gradient Echo, 5 × k-t Broad-use Linear Acquisition Speed-up Technique, 11 training profiles, 1.31 × 1.32 × 10 mm^3^ acquired resolution, pre-pulse delay 100 ms, acquisition shot 123 ms/slice, three short axis slices) [48]. Intravenous adenosine will be administered at 140 mcg/kg/min for three minutes under continuous ECG monitoring. Adequate hemodynamic response is assessed by either i) heart rate increase by ≥10%, ii) systolic blood pressure decrease of ≥10 mmHg or iii) symptoms attributed to adenosine administration. If there is inadequate hemodynamic response then the dose will be increased to 170 and then to 210 μg/kg/min for a further two minutes until hemodynamic response is achieved. The contrast injection will be performed using a dual-bolus technique, by intravenous route in the ante-cubital fossa, of 0.1 mmol/kg of gadolinium-DTPA (diethylene triamine pentaacetic acid) (gadopentetate dimeglumine; Magnevist, Bayer, Berlin, Germany) for the main bolus, preceded by the same volume of a 10% dilute contrast agent dose for the pre-bolus, both administered at a rate of 4.0 ml/s, followed by a saline flush using a using a power injector (Spectris, Solaris, Pennsylvania, United States) [49].
4. Resting wall motion and LV function. Cine image stack covering the entire heart in the LV short axis plane at one slice per breath-hold in end-expiration and parallel to the mitral valve annulus (bSSFP, multiphase, 10 to 12 contiguous slices, spatial resolution 2.0 × 1.63 × 8 mm^3^ and 30 cardiac phases) [50, 51].
5. Tissue tagging for strain analysis and diastology. Spatial modulation of magnetization pulse sequence (spatial resolution 1.51 × 1.57 × 10 mm^3^, tag separation 7 mm, ≥18 phases, typical TR/TE 5.8/3.5 ms and flip angle 10°).
6. Aortic distensibility. Cine images of the ascending aorta (50 phases) at the level of the PA bifurcation and the descending aorta, transverse to the vessel according to Lee *et al*. [35]. For aortic stiffness, blood pressure and heart rate are recorded immediately prior to the multi-phase SSFP cine image (24 phases).
7. Resting first-pass myocardial perfusion study. Pulse sequence, slice positioning and injection characteristics identical to the stress perfusion scan as above in step 3.
8. Late gadolinium enhancement (LGE). Performed between 10 and 15 minutes after step 7. Inversion recovery-prepared T1-weighted gradient echo. The optimal inversion time to null signal from normal myocardium will be determined using a modified Look-Locker approach [52]. Typical parameters: TE 2.0 ms, TR 3.5 ms, flip angle 25°, acquired spatial resolution 1.54 × 1.76 × 10 mm^3^. Inversion time adjusted according to variable TI scout. Alternate heart beat acquisitions by navigator is an option for poor breath holders. Performed in 10 to 12 short axis slices with further slices acquired in the vertical and horizontal long axis orientations, or phase-swapped, if indicate based on LGE imaging obtained, wall-motion or perfusion defects.
9. Post-contrast T1 mapping 15 minutes following last contrast injection at step 7. Acquisition and slice positioning as above in step 2.

T1 mapping, tissue tagging and perfusion imaging are performed in three identical short-axis positions. These will be determined using the ‘three-of-five’ approach by acquiring the central three slices of five parallel short-axis slices spaced equally from mitral valve annulus to LV apical cap [53].

### CMR image analysis and reporting

Image analysis will be performed offline, blinded to patient characteristics and treatment arm, using commercially available software (cvi42 version 4.1.3, Circle Cardiovascular Imaging Inc., Calgary, Canada and inTag version 1.0, CREATIS lab, Lyon, France) according to international standards for reporting of CMR studies [54].

LV volume and EF will be calculated from the short axis cine-stack using standard criteria to delineate cardiac borders [54]. Regional wall motion in 17 cardiac segments will be graded visually. Aortic cross sectional measurements will be made by manual planimetry of the endovascular-blood pool interface, at the times of maximal and minimal distension of the aorta. Aortic distensibility, compliance and stiffness index are calculated by standard methods using blood pressure measurements taken at the time of image acquisition with formulas and definitions listed in Table 2 [55].

**Table 2:** **Definitions and formulas of parameters used in the assessment of arterial stiffness**

Parameter	Definition	Formula
Aortic Compliance	The absolute change in vessel diameter (or area) for a given change in pressure	ΔD/ΔP
Aortic Distensibility	The absolute change in vessel diameter (or area) for a given change in pressure	ΔD/(ΔP × D)
Stiffness Index	The ratio of the natural logarithm of SBP/DBP to the relative change in diameter	ln(Ps/Pd)/((Ds-Dd)/Dd)

Δ; change in; D, diameter; d, diastole; ln, natural logarithm; P, pressure; s, systole. Adapted from Oliver and Webb [55].

Native and post-contrast myocardial T1 will be measured [56]. Care will be taken to ensure a conservative region of interest and to avoid partial-volume effects from neighboring tissue or blood pool. Regions of interest are manually motion-corrected as required. The reciprocal of T1 is calculated as R1. ECV is calculated using the following equation [57]:
1

Where hct is the hematocrit. Myo pre and myo post are the pre-contrast and post-contrast myocardial T1 values. Blood pre and blood post are the pre-contrast and post-contrast blood pool T1 values. Strain analysis will use data from the tagged cine series. Endocardial and epicardial contours are drawn by a semi-automated process for each slice. Peak circumferential systolic strain and rotation will be calculated for the three short axis slices at the level of apex, mid-ventricle and base. LV twist is calculated by subtracting the basal rotation from the apical rotation. The method of determining torsion takes the radius and length of the heart into account, describing the torsion as the circumferential-longitudinal shear angle. This makes the measurement comparable between hearts of different sizes and is related to fiber orientation and processes in the myocardium [58, 59]. Basal and apical radius is calculated from measuring area by epicardial contours on cine imaging in diastole at the same slice location as the tagged images. Base-to-apex length is determined by subtracting the slice locations. The equation used to determine torsion is:
2

Myocardial perfusion will be assessed by visual comparison of stress and rest CMR perfusion scans (16 segments of the modified 16 segment American Heart Association/American College of Cardiology model) [60] with scores of 0 (normal), 1 (equivocal), 2 (non-transmural ischemia <50%), 3 (non-transmural ischemia ≥50%) or 4 (transmural ischemia). In addition, quantitative MBF estimates will be obtained using Fermi-constrained deconvolution, or other methods and myocardial perfusion reserve (MPR) calculated by dividing stress by rest MBF values [38].

LGE images will be analyzed visually by two experienced observers and any relevant patterns of enhancement are described based on a 17-segment model with scores of 0 (no hyperenhancement), 1 (1 to 25% mural thickness), 2 (26 to 50% mural thickness), 3 (51 to 75% mural thickness) or 4 (>75% mural thickness) allocated to each segment. Quantitative analysis of LGE will also be performed. LGE volume will be calculated across the whole LV stack by the modified Simpson’s method. To avoid confounding for artifacts, a conservative threshold for LGE is employed at five SDs from remote, normal myocardium. The amount of LGE will be presented as a percentage against normal myocardium.

### Reproducibility

CMR measurements have been validated in previous reproducibility studies. In our hands, the inter- and intra-observer reproducibility for measurement of aortic distensibility by CMR is excellent. In a clinical study of 49 volunteers, the intra-observer mean difference for diastolic (minimum) aortic volume was 0.009 ± 0.039 ml and the mean difference for systolic (maximum) aortic volume was 0.0075 ± 0.039 ml (*P* = not significant). The coefficient of variation (CoV) in the diastolic and systolic measurements were 1.4% and 1.1%, with an intra-class correlation coefficient (ICC) of r = 0.998 and r = 0.998, respectively [61]. Analysis of tissue-tagged CMR images shows an intra-observer CoV for circumferential strain of 4.3%, and 1.2% for LV twist (n = 12). The inter-study CoV of circumferential strain is 3.7% and 9.6% for LV twist. The ICC shows excellent intra-observer, inter-observer and inter-study reproducibility of circumferential strain, ranging from 0.95 to 0.98. The ICC suggested excellent intra-observer and inter-observer reproducibility (0.97 and 0.95, respectively) of LV twist and good inter-study reproducibility of LV twist (0.67) [62]. Quantitative perfusion analysis has an intra-observer CoV of 13 to 18% and an inter-observer CoV of 8 to 15%. In a pilot study of 11 volunteers, the inter-observer mean difference was 0.22 ± 14.82% to 4.53 ± 12.83%, and the intra-observer mean difference was 4.51 ± 13.22% to 7.78 ± 20.19% [63]. The inter- and intra-observer ICC of quantitative perfusion by CMR has been previously shown to be 0.83 and 0.80, respectively [64]. In this study, repeated measurements of 12 randomly selected scans, with blinding to the original measurements, will be performed for reproducibility analysis.

### Biomarkers

As part of the exploratory objectives, CADERA will enable linkage of biomarkers to CMR measurements of CVD. Specifically, the following will be clinically evaluated: rheumatoid factor (RF), ACPA, CRP, ESR, lipid profile, high-sensitivity CRP, serum amyloid A, fibrinogen, adiponectin, interleukin-6, TNF, intercellular adhesion molecule-1, vascular cellular adhesion molecule 1, CD40 ligand and N-terminal prohormone of B-type natriuretic peptide.

### Annual follow-up and the Inflammatory Arthritis disease CONtinuum (IACON) study

Created in 2010 at the NIHR LMBRU, the IACON (Inflammatory Arthritis disease CONtinuum) study is a major longitudinal cohort study in inflammatory arthritis. This facilitates collection of CVD outcome measurements in patients with inflammatory arthritis at Leeds from disease inception onwards. On completion of the study, all CADERA study patients will enter IACON, permitting continued follow-up annually or as clinically indicated. There is no fixed endpoint for data collection and study duration of IACON. CMR findings will be linked to clinical outcome through long-term follow-up in this registry.

### Safety and adverse events

CMR is a standard clinical imaging modality in everyday clinical use and risks to the study participants are small. Adenosine stress agents carry a small risk of adverse effects including transient atrio-ventricular block and bronchospasm. CMR contrast agents carry a low risk of allergic reactions (approximately 1:10,000). To avoid the development of nephrogenic systemic fibrosis relating to some CMR contrast agents, patients with renal failure and an eGFR of less than 30 ml/min/1.73 m^2^ will not be recruited. All serious adverse events that occur as a result of the CMR will be reported without formal statistical testing being undertaken.

## Discussion

Early diagnosis and immediate treatment of new, onset, treatment-naïve RA is crucial to ensure the best possible treatment outcomes. Studies demonstrate TNFi agents confer additional structural benefit but, in particular, may be able to modulate disease progression in a proportion of patients. It remains unclear whether use of non-bDMARD (MTX) impedes this potential effect. We postulate with the VEDERA study that first-line TNFi therapy is qualitatively and quantitatively superior, with better clinical, structural and immunological outcomes when compared with non-biological DMARDs. The bolt-on CADERA study will provide a comprehensive CV evaluation of the VEDERA population to assess the prevalence and severity of CVD in a treatment-naïve patient population of new-onset RA with comparison to clinical parameters, such as RA disease severity. The study will also evaluate whether effective RA disease control (remission) can improve CVD as assessed by CMR and, importantly, whether achieving remission through first-line TNFi offers any additional benefit over initial synthetic DMARD-induced remission. With linkage of CMR assessment, CVD biomarkers and long-term outcomes with follow-up in the IACON registry, we hope to improve our understanding of the pathophysiology of CVD in the RA population. The knowledge gained from these studies may contribute towards more effective use of targeted therapies for patients with RA and improve long-term health-economic benefits.

## Trial status

This trial is ongoing. Patient recruitment and follow-up is underway. Recruitment began in February 2012 and is expected to end in June 2015.

## Abbreviations

ACPA: Anti-citrullinated peptide antibody
ACR: American College of Rheumatology
AHA: American Heart Association
bDMARD: Biological disease modifying anti-rheumatic drug
bSSFP: Balanced steady-state free precision
CAD: Coronary artery disease
CADERA: Coronary Artery Disease Evaluation in Rheumatoid Arthritis trial
CIMT: Carotid intimal-media thickness
CMR: Cardiac magnetic resonance
CoV: Coefficient of variability
CRP: C-reactive protein
CV: Cardiovascular
CVD: Cardiovascular disease
DAS: Disease activity score
DMARD: Disease modifying anti-rheumatic drug
ECG: Electrocardiogram
ECV: Extra-cellular volume
EF: Ejection fraction
ESR: Erythrocyte sedimentation rate
ETN: Etanercept
EULAR: The European league against rheumatism
IACON: Inflammatory Arthritis disease CONtinuum study
LGE: Late gadolinium enhancement
LMBRU: Leeds Musculoskeletal Biomedical Research Unit
LV: Left ventricle
MBF: Myocardial blood flow
MOLLI: Modified Look-Locker inversion
MTX: Methotrexate
PET: Positron emission tomography
RA: Rheumatoid arthritis
RF: Rheumatoid factor
SD: Standard deviation
SPECT: Single-photon emission computed tomography
TE: Echo time
TNF: Tumor necrosis factor
TNFi: Tumor necrosis factor inhibitor
TR: Repetition time
TTE: Transthoracic echocardiography
VEDERA: Very Early versus Delayed Etanercept in Rheumatoid Arthritis trial.
